# Diagnosis and Management of Radiation Necrosis in Patients With Brain Metastases

**DOI:** 10.3389/fonc.2018.00395

**Published:** 2018-09-28

**Authors:** Balamurugan Vellayappan, Char Loo Tan, Clement Yong, Lih Kin Khor, Wee Yao Koh, Tseng Tsai Yeo, Jay Detsky, Simon Lo, Arjun Sahgal

**Affiliations:** ^1^Department of Radiation Oncology, National University Cancer Institute, National University Health System, Singapore, Singapore; ^2^Department of Pathology, National University Hospital, Singapore, Singapore; ^3^Department of Diagnostic Imaging, National University Hospital, Singapore, Singapore; ^4^Nuclear Medicine, Advanced Medicine Imaging, Singapore Institute of Advanced Medicine Holdings, Singapore, Singapore; ^5^Department of Neurosurgery, National University Hospital, Singapore, Singapore; ^6^Department of Radiation Oncology, Sunnybrook Health Sciences Center, University of Toronto, Toronto, ON, Canada; ^7^Department of Radiation Oncology, University of Washington School of Medicine, Seattle, WA, United States

**Keywords:** brain metastases (BM), stereotactic radiosurgery, whole brain radiation therapy, radiation necrosis, MRI imaging techniques

## Abstract

The use of radiotherapy, either in the form of stereotactic radiosurgery (SRS) or whole-brain radiotherapy (WBRT), remains the cornerstone for the treatment of brain metastases (BM). As the survival of patients with BM is being prolonged, due to improved systemic therapy (i.e., for better extra-cranial control) and increased use of SRS (i.e., for improved intra-cranial control), patients are clinically manifesting late effects of radiotherapy. One of these late effects is radiation necrosis (RN). Unfortunately, symptomatic RN is notoriously hard to diagnose and manage. The features of RN overlap considerably with tumor recurrence, and misdiagnosing RN as tumor recurrence may lead to deleterious treatment which may cause detrimental effects to the patient. In this review, we will explore the pathophysiology of RN, risk factors for its development, and the strategies to evaluate and manage RN.

## Introduction

Radiotherapy is the cornerstone management for BM. Historically, WBRT was the only available modality for management. Although it provides palliation of symptoms, the survival of patients treated with WBRT alone remains poor (1, 2). Technological advancements have now made SRS widely available, and the effectiveness of SRS in controlling BM is well-documented (3).

Often a combination of these two approaches are used, either upfront or as salvage. Prior randomized controlled trials have shown that the addition of SRS to WBRT improves the local intra-cranial control and survival for patients with a single brain metastasis (4, 5). In contrast, patients treated with SRS (without WBRT), have a higher risk of distant intra-cranial relapse, but no detriment in survival (3). Therefore, National Comprehensive Cancer Network guidelines recommend that patients undergo routine surveillance MRI imaging every 2–3 monthly, especially if treated with SRS alone^1^. Often, treatment-related changes, detected on follow-up scans, are indistinguishable from tumor recurrence. This creates a diagnostic dilemma for many clinicians, as the management of each are vastly different. One of the feared complications of BM treatment is symptomatic RN; this often affects patient quality-of-life and can lead to significant morbidity.

In this review, we will explore the pathophysiology of RN, risk factors for its development, and the strategies to evaluate and manage RN.

## Incidence of RN

Within the context of BM, the true incidence of RN is hard to estimate and probably lies between 5 and 25% (6–10). The definition of RN varies across studies, and only some required histological confirmation. Moreover, the wide variation may be attributable to improved quality and frequency of diagnostic imaging, increased awareness (leading to better reporting) within the oncology community and length of follow-up. For example, a study by Chin et al. where pathological confirmation or temporal resolution was required, the incidence was reported to be 7% (8). In contrast, using primarily imaging-based diagnosis, Minniti et al. reported a 24% incidence of RN (14% symptomatic, 10% asymptomatic), for which they relied on imaging features, such as increased contrast enhancement, non-progression of lesion over 4 months and reduced perfusion on dynamic MRI sequences (6).

## Pathophysiology of RN

Early experiments were done on rats (11) and dogs (12) with single-fraction brain radiation(10–25 Gy). These experiments showed that the radiation tolerance of the brain was intricately linked to dose, volume of treatment and was a function of time elapsed since radiation. Histopathological analysis from these animal experiments demonstrated changes in vasculature, as well as demyelination, in the irradiated areas. Higher doses consistently led to demyelination and necrosis, as well as an earlier manifestation of necrosis.

There are two theories behind the pathophysiology of RN, however it is likely that the true cause is multi-factorial (13).

1. Vascular injury theory
  - Radiation disrupts the blood-brain barrier, resulting in increased capillary leakiness and vascular permeability (14). Radiation, especially in large fraction sizes >8 Gy, activates acid sphingomyelinase and causes upregulation of ceramide, which in turn causes endothelial apoptosis (15). This leads to increased oxygen-free radicals, a pro-inflammatory milieu (through release of Tumor-necrosis factor and interleukin-1 beta) (16, 17), increased production of vascular-endothelial growth factor (VEGF) (18) and intercellular adhesion molecule (ICAM-1) (19). This cascade leads to vessel narrowing and fibrinoid necrosis of small vessels resulting in ischemia and cell death (20).
2. Glial cell theory
  - Radiation can also damage glial cells. Damage to oligodendrocytes and their progenitors result in demyelination (21). Hypoxia caused by endothelial cell damage leads to liberation of hypoxia-inducible factor 1α and VEGF. VEGF induces neo-angiogenesis, but these tend to be leaky capillaries; resulting in perilesional edema and contrast extravasation.

## Risk factors for RN and mitigation strategies

A direct cause-effect relationship for RN is hard to establish, but many risk factors have been identified. These include tumor volume, prescribed dose, fraction size, volume of normal brain irradiated, previous use of radiation and the use of concurrent systemic therapy (22). Many of these risk factors were established in patients being treated for arterio-venous malformations and gliomas, but can be extrapolated to BM.

1. Dose-volume interplay
  - Early studies from RTOG 90-05 recommended the maximum safe radiation dose to be based on tumor volume (23). The 12-months cumulative incidence of RN was 8%, with larger tumors having increased rates of RN. For example, lesions below ≤ 20 mm were safely treated with 24 Gy, 21–30 mm with 18 Gy and 31–40 mm with 15 Gy. However, this data is based on a mixture of recurrent primary and secondary brain tumors, and all patients had prior radiation.
  - For patients undergoing SRS (with or without WBRT), the volume of brain parenchyma receiving higher than 10 or 12 Gy (V10 and V12, respectively) has been correlated to RN. Blonigen et al reported that the risk of RN is higher when V10 > 10.5 cm^3^ or V12 > 7.9 cm^3^ (9). The use of V10 and V12 corroborates with studies in AVM (24) and other intracranial tumors (25). It remains unclear how this volume should be defined, in particular if the gross tumor volume should be excluded from normal brain parenchyma. Fractionated stereotactic radiotherapy has been proposed to mitigate this risk, but strong comparative evidence is still lacking (26, 27).
2. Prior radiation exposure
  - The use of prior WBRT or SRS and the time interval between re-irradiation influences the risk of RN. For example, the risk of RN with SRS in the setting of prior SRS (to the same lesion) was reported to be 20% at 1 year, 4% when prior WBRT had been used and 8% when concurrent WBRT is used (22). The risk was reported to be 3% when no prior irradiation had been given (22). In the setting of prior WBRT, it is unclear if the fraction size of WBRT influences the risk.
3. Chemotherapy
  - The use of chemotherapy in the setting of primary brain tumors increases the risk of RN (28). Within the context of BM, the use of capecitabine within 1 month of SRS appeared to increase the risk of RN (22).
4. Location
  - Extrapolating from AVM studies, certain locations within the brain may have higher risk of RN. The frontal cortex appears to carry the highest risk for RN while the brainstem is more resistant to developing RN (24).
  - Japanese investigators suggest that superficial lesions are at a lower risk of RN, because of the dose spillage to extraparenchymal tissue (skull vault, skin, etc.) (29).
5. Histology
  - Miller et al suggest certain histological subtypes to have a higher risk of RN (30). These include renal carcinoma, lung adenocarcinoma (ALK rearrangement specifically), HER2-amplied breast cancer, and BRAF V600 wild-type melanoma.
6. Planning Target Volume (PTV) margin
  - While a larger GTV (gross tumor volume) to PTV margin would allow for setup and positional uncertainties, the consequence is that target volume increases significantly and larger volume of normal brain parenchyma is included in the prescription isodose. In a randomized trial, comparing 1 and 3 mm GTV-PTV expansion, the local control was similar in both groups, however the 3 mm group had a higher incidence of biopsy-proven RN (12.5 vs. 2.5%, *p* = 0.1) (31). Although clinically significant, statistical significance may not have been reached due to the low patient number.
7. Intrinsic radiosensitivity
  - Data from AVM treatment suggest that patients who developed RN had an increased sensitivity to radiation. This was demonstrated using survival curves (*in vitro*) from skin fibroblasts obtained from patients who developed RN (32). Although intrinsic radiosensitivity may be a risk factor, there are no practical methods to quantify this in the clinics.

## Diagnosis and investigations for patients with suspected RN

• Imaging

Magnetic resonance (MR) imaging is the most commonly used modality to investigate RN. However, the imaging features of radiation necrosis and tumor recurrence overlap considerably, with both entities demonstrating some degree of contrast enhancement and perilesional edema (33, 34). Most of the time, there is a combination of both entities (35).

Temporal changes alone (i.e., increase in size over time) is not specific to either entity. While certain enhancement patterns described in the literature as “Swiss cheese,” “soap bubble,” or “cut green pepper” were initially thought to favor radiation necrosis, these have only a 25% positive predictive value (36). Dequesada et al. noted that gyriform lesions and edema with marginal or solid enhancement suggested at least some viable tumor, adding that a lesion quotient (LQ) (which is the ratio of the nodule on T2 sequence to the total enhancing area on T1 sequence) of >0.6 was suggestive of tumor recurrence, while an LQ of < 0.3 favored radiation necrosis alone (36). Other authors however found this feature to be only 8% sensitive (37).

In practice, the low predictive value of conventional MR features prompted the need for more advanced tools, such as MR spectroscopy (MRS), MR perfusion, and Positron Emission Tomography (PET) to help increase diagnostic confidence. These three advanced techniques are discussed below.

### MR perfusion

Viable tumor has intact vasculature and thus higher perfusion and blood volume than necrotic tissue. An increased relative cerebral blood volume (rCBV) based on dynamic susceptibility-weighted MRI has been used for differentiating tumor from necrosis (38–40). Unfortunately, published data have been inconsistent. Hu et al reported rCBV of < 0.71 as 92% sensitivity and 100% specificity for radiation necrosis, while another suggested a rCBV cutoff of < 2.1 (100% sensitivity and specificity) (38, 41). Barajas et al reported significant overlap in rCBV values and proposed using the percentage of signal-intensity recovery (PSR) (33). Furthermore, rCBV values vary between machines, depend on the acquisition methods and are confounded by signal-intensity pileup artifacts, and susceptibility artifacts from blood and contrast pooling within the lesions. Intravoxel incoherent motion (IVIM) is another method that provides quantitative diffusion and perfusion measurements based on a diffusion-weighted imaging (DWI) MR acquisition. IVIM has been shown to be superior to rCBV for distinguishing recurrent tumor from RN (42) and has been validated against gold standard histopathology (35).

### MR spectroscopy

Assessment of the metabolite composition within BM is another useful method that has published threshold values. Increased choline-creatinine (Cho:Cr) and choline–N-acetyl aspartate (Cho:NAA) ratios may favor tumor recurrence (43). Zeng et al found that when both Cho:Cr and Cho:NAA were above 1.71, sensitivity, specificity and diagnostic accuracy were 94.1%, 100%, and 96.2%, respectively (44). In contrast, an elevated lipid-lactate peak and generalized decrease in other metabolites supported radiation necrosis (45). MRS is limited by voxel size, often requiring the lesion to be larger than 1 cm^3^, and is also affected by sampling errors within heterogeneous tumors. Chemical exchange saturation transfer (CEST) is a novel method that is sensitive to mobile proteins and peptides and has shown early promise as well in identifying recurrent tumor after SRS (46).

### PET-CT

PET imaging has better spatial resolution and coverage than MRS and use of Fluorodeoxyglucose (FDG) PET in this clinical setting was first proposed in 1982, relying on the presumed increased glucose metabolism in tumors (47). However, multiple studies have shown FDG-PET unhelpful for diagnosing RN (48, 49). Amino acid tracers then became particularly useful in PET imaging because of high amino acid utilization in tumors for cell proliferation and extracellular matrix production (50). Moreover, normal brain tissue has relatively lower amino acid uptake, and this provides good tissue contrast. Tracers including Carbon-11 methionine (MET), Fluoro-l-thymidine (FLT) and Fluoroethyltyrosine (FET) have been used with promising results (51, 52, 53, 54). Of particular interest is FET-PET, where the addition of dynamic data analysis reported a sensitivity of 100% and specificity of 93%, comparable to some MRS results (55).

**Figure 1:**
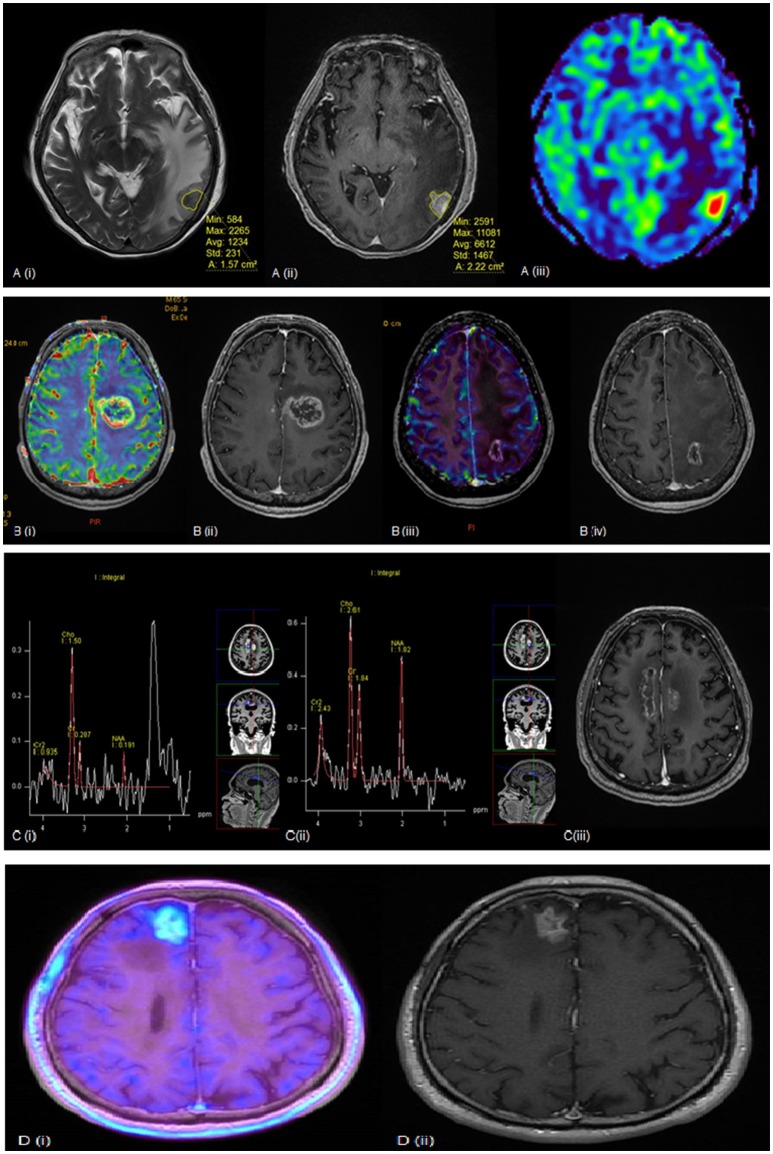
**(A)** (i) T2 weighted (ii) post-contrast T1 weighted and (iii) rCBV MR perfusion sequences of a lesion seen within the left temporal lobe. The lesion quotient is calculated using the ratio of the hypointense nodule on T2W imaging to the total enhancing area on T1W imaging. This case showed a lesion quotient of 0.71 and increased rCBV is suggestive of tumor recurrence. **(B)** (i) rCBV and (ii) post-contrast T1 weighted sequences showing increased blood flow within the periphery of the lesion. This was a tumor recurrence proven by histopathology. (iii) rCBV and (iv) post-contrast T1W sequences of another patient showing no increased blood flow within the periphery in keeping with radiation necrosis. **(C)** (i, ii) MR spectroscopy and (iii) post-contrast T1 weighted sequences of a growing pericallosal lesion post-WBRT. (i) typical high lipid-lactate peak seen in radiation necrosis at the right cingulum while (ii) shows increased Cho:Cr and Cho:NAA ratios suggestive of tumor recurrence over the left cingulum. **(D)** (i) F-18 FET PET showing intense amino acid tracer uptake within the enhancing lesion seen in (ii) post-contrast T1 weighted sequence. This is suggestive of tumor recurrence and found to be recurrent RCC metastasis on histology.

Figure 1 illustrates several examples where the above modalities have been used to evaluate RN. For now, there is no single modality that has been shown to accurately differentiate tumor recurrence from radiation necrosis, and biopsy is still regarded as the diagnostic gold standard. In view of the limitations of each modality, a multi-modality approach may be warranted to improve diagnostic confidence.

• Pathological assessment

**Figure 2:**
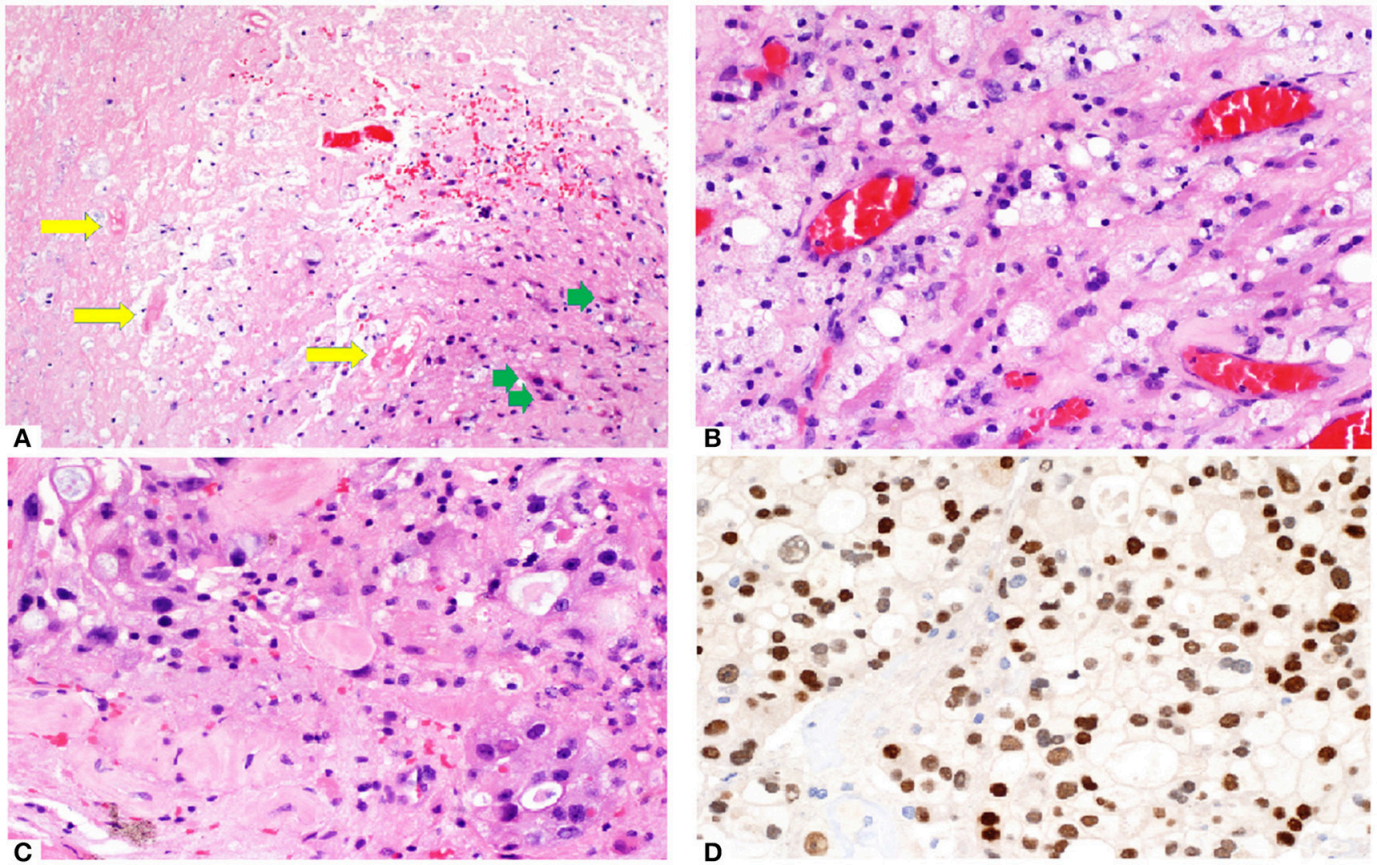
**(A)** Brain tumor resection specimen from a patient with known metastatic breast carcinoma 6 months after Gamma knife SRS (20 Gy to 50% isodose line). The area of necrosis appears hypocellular and sharply demarcated from the surrounding gliotic brain. Few necrotic, hyalinized blood vessels (yellow arrows) are present, as well as scattered reactive astrocytes (green arrows). Overall features are those of a radiation necrosis. **(B)** Foamy macrophages are often present. The capillaries appear ectatic and congested. **(C)** Focal area shows increased cellularity with more nuclear pleomorphism in an otherwise hyalinised background, raising the possibility of residual viable tumor. **(D)** Immunostain (brown) with GATA3 labels numerous viable tumor cells. Nuclear pleomorphism appears more prominent and highlighted by this nuclear stain.

Histopathology from surgically resected lesions after SRS commonly shows a mix of residual tumor cells and RN (56–60). Endothelial cells, which are most susceptible to radiation damage, often manifest with fibrinoid necrosis, hemorrhage, hyalinization and thrombosis of the blood vessels, resulting in hypoxic injury to the surrounding tissue (61). The area of necrosis is usually paucicellular, surrounded by highly gliotic brain tissue consisting of GFAP-reactive astrocytes demonstrating prominent cytoplasmic ramification. Foamy macrophages and hemosiderophages are often encountered, occasionally with dystrophic calcification. In addition, radiation-induced cytologic atypia maybe seen, featuring cytomegaly with bizarre “bubbly” nuclei, maintaining an overall low nuclei-cytoplasmic ratio. In contrast, in recurrent tumor, tumor necrosis often appears cellular with ghost-outline of the tumor cells, demonstrating high nucleo-cytoplasmic ratio. Careful examination of the blood vessels is important as residual viable tumor maybe present around the Virchow Robin spaces or as intravascular clusters, reminiscing the hematogenous route taken by the tumor. In the setting of suspected tumor recurrence with superimposed radiation-induced damage, a limited panel of immunohistochemistry, depending on the known primary tumor types, can be helpful in highlighting the viable tumor which may not be obvious on hematoxylin-eosin stained slides. Histo-pathological assessment of a patient with RN is shown in Figure 2.

## Management of RN

The management of RN primarily depends on the presence of symptoms. Symptomatic patients may experience headaches, nausea, cognitive impairment, seizures or focal deficits relating to the location of the lesion.

Data from patients with nasopharyngeal carcinoma with radiological RN suggest that one third or less of patients have spontaneous regression over time, and that it is not always an irreversible progressive process (62). As such, observation is a viable treatment option for small and/or asymptomatic RN. However, closer clinical and radiological monitoring is warranted (e.g., every 6–8 weeks, and then extending to 12–16 weeks once the lesion is stable/regressed). Patel et al. reported that approximately one-third of patients treated with SRS have increase in lesional size during follow-up (63), occurring between 6 weeks and 15 months post-SRS. Counterintuitively, patients with lesion progression had the longest survival compared to patients with stable or decreased lesional size. They hypothesized that post-SRS lesional growth may be due to brisk reactive immune response, rather than tumor recurrence. However, this has to be interpreted with caution, as there is an inherent selection bias.

For symptomatic patients, oral corticosteroids (such as dexamethasone) is the preferred first line. Corticosteroids reduce the inflammatory signals and cytokines produced by the necrotic tissue and reduce the leakiness of the blood-brain barrier (64). Due to resolution of the edema, most patients experience rapid improvement once steroids are initiated. There are no studies guiding the dose of steroids. In our practice, we prefer to use dexamethasone (4–8 mg per day), with a gradual taper of the dose. Unfortunately, many patients will require steroids for a long duration and are subject to steroid-toxicity, such as myopathy, iatrogenic Cushing's syndrome, gastric ulcers etc.

As VEGF has been shown to be a key mediator in RN (65), there is considerable interest in the use of bevacizumab (humanized monoclonal antibody against VEGF) to treat steroid-refractory RN. A pooled analysis involving 71 patients, showed that the use of bevacizumab had a radiographic response rate of 97% and clinical improvement rate of 79% with a mean decrease in dexamethasone of 6 mg (66). The median decrease in FLAIR signal and enhancing-volume was ~60%. One small randomized study has been performed using bevacizumab for RN that allowed a cross-over from the placebo group (67). All 14 patients eventually ended up receiving bevacizumab, and all patients showed radiographic response. No differences could be demonstrated in symptomatology, however the majority of the patients on dexamethasone were able to reduce their doses. As such, bevacizumab appears to be a promising agent; however, the durability of response and toxicities associated with bevacizumab, such as hemorrhage, thrombosis and impaired wound healing must be taken into account.

Anticoagulants and medications which moderate perfusion have been tested in RN, but are not routinely used. For example, oral pentoxifylline and vitamin E were evaluated in 11 patients, with their MRI FLAIR volume changes recorded over time (68). Although there was an overall average decrease in edema, some patients had an increase in edema.

In another study, heparin and warfarin were evaluated in eight patients, with slightly over half showing some functional recovery (69). However, it is unclear if anticoagulation needs to be continued indefinitely.

Hyperbaric oxygen therapy (HBOT) is designed to promote perfusion and angiogenesis. The use of HBOT in RN is mostly limited to case reports where the efficacy is not well-documented (70–72). Investigators have also studied the use of HBOT as prophylaxis, and have shown promising results (73). However, HBOT is expensive, requires specialized facilities and involves a significant time commitment with prescribed treatment ranging from 20 to 40 sessions.

For patients who remain symptomatic despite conservative management, or in whom there is diagnostic uncertainty, surgical resection can be considered. The main advantages of surgical resection are relief of any mass effect and histological confirmation. This influences subsequent treatment decisions and can help with prognostication. Removing the nidus of necrotic tissue responsible for the peri-lesional edema will provide patients symptomatic relief, and allow weaning off steroids. Patient selection remains an important consideration, and includes surgical accessibility, overall performance status and life expectancy. Early reports suggest a high risk of morbidity with surgical resection, but it remains to be seen if these risks still persist in the modern era (74). In situations where there is necrotic tissue admixed with viable tumor, clinical judgment is required to decide on further management.

Novel techniques, such as laser interstitial thermal therapy (LITT) are emerging as treatment options. LITT is an image-guided approach which generates high temperatures using a laser fiber, and facilitates ablation of both tumor tissue, or VEGF-producing reactive glial cells (75). A prospective study has shown this to be safe and allow weaning of steroids in a third of patients (76).

## Conclusion

RN will be increasingly encountered due to the widespread use of SRS. Symptomatic RN can cause significant morbidity and should be managed pro-actively. There is no single modality which can reliably distinguish RN from recurrent tumor, and a multi-modal approach is often required. For patients with symptomatic RN, oral corticosteroid therapy and bevacizumab are both effective. A minority of patients, with an unclear diagnosis, or refractory symptoms, will require surgical resection. As RN proves to be a challenging condition to diagnose and manage, risk factor mitigation becomes important in clinical decision making.

## Author contributions

BV and AS substantial contributions to the conception or design of the work. BV, CT, CY, and JD drafting the work or revising it critically for important intellectual content. All authors provide approval for publication of the content. All authors agree to be accountable for all aspects of the work in ensuring that questions related to the accuracy or integrity of any part of the work are appropriately investigated and resolved.

### Conflict of interest statement

The authors declare that the research was conducted in the absence of any commercial or financial relationships that could be construed as a potential conflict of interest.

## Footnotes

^1^https://www.nccn.org/professionals/physician_gls/pdf/cns.pdf (Accessed June 2018).

## References

[1] Borgelt B Gelber R Kramer S Brady LW Chang CH Davis LW. The palliation of brain metastases: final results of the first two studies by the Radiation Therapy Oncology Group. Int J Radiat Oncol Biol Phys. (1980) 6:1–9. 10.1016/0360-3016(80)90195-9 6154024

[2] Mulvenna P Nankivell M Barton R Faivre-Finn C Wilson P McColl E. Dexamethasone and supportive care with or without whole brain radiotherapy in treating patients with non-small cell lung cancer with brain metastases unsuitable for resection or stereotactic radiotherapy (QUARTZ): results from a phase 3, non-inferiority, randomised trial. Lancet (2016) 388:2004–14. 10.1016/S0140-6736(16)30825-X 27604504 PMC5082599

[3] Sahgal A Aoyama H Kocher M Neupane B Collette S Tago M. Phase 3 trials of stereotactic radiosurgery with or without whole-brain radiation therapy for 1 to 4 brain metastases: individual patient data meta-analysis. Int J Radiat Oncol Biol Phys. (2015) 91:710–7. 10.1016/j.ijrobp.2014.10.024 25752382

[4] Andrews DW Scott CB Sperduto PW Flanders AE Gaspar LE Schell MC. Whole brain radiation therapy with or without stereotactic radiosurgery boost for patients with one to three brain metastases: phase III results of the RTOG 9508 randomised trial. Lancet (2004) 363:1665–72. 10.1016/S0140-6736(04)16250-8 15158627

[5] Kondziolka D Patel A Lunsford LD Kassam A Flickinger JC. Stereotactic radiosurgery plus whole brain radiotherapy versus radiotherapy alone for patients with multiple brain metastases. Int J Radiat Oncol Biol Phys. (1999) 45:427–34. 10.1016/S0360-3016(99)00198-4 10487566

[6] Minniti G Clarke E Lanzetta G Osti MF Trasimeni G Bozzao A. Stereotactic radiosurgery for brain metastases: analysis of outcome and risk of brain radionecrosis. Radiat Oncol. (2011) 6:48. 10.1186/1748-717X-6-48 21575163 PMC3108308

[7] Kohutek ZA Yamada Y Chan TA Brennan CW Tabar V Gutin PH. Long-term risk of radionecrosis and imaging changes after stereotactic radiosurgery for brain metastases. J Neurooncol. (2015) 125:149–56. 10.1007/s11060-015-1881-3 26307446 PMC4726630

[8] Chin LS Ma L DiBiase S. Radiation necrosis following gamma knife surgery: a case-controlled comparison of treatment parameters and long-term clinical follow up. J Neurosurg. (2001) 94:899–904. 10.3171/jns.2001.94.6.0899 11409517

[9] Blonigen BJ Steinmetz RD Levin L Lamba MA Warnick RE Breneman JC. Irradiated volume as a predictor of brain radionecrosis after linear accelerator stereotactic radiosurgery. Int J Radiat Oncol Biol Phys. (2010) 77:996–1001. 10.1016/j.ijrobp.2009.06.006 19783374

[10] Varlotto JM Flickinger JC Niranjan A Bhatnagar AK Kondziolka D Lunsford LD. Analysis of tumor control and toxicity in patients who have survived at least one year after radiosurgery for brain metastases. Int J Radiat Oncol Biol Phys. (2003) 57:452–64. 10.1016/S0360-3016(03)00568-6 12957257

[11] Calvo W Hopewell JW Reinhold HS Yeung TK. Time- and dose-related changes in the white matter of the rat brain after single doses of X rays. Br J Radiol. (1988) 61:1043–52. 10.1259/0007-1285-61-731-1043 3208008

[12] Benczik J Tenhunen M Snellman M Joensuu H Farkkila M Joensuu R. Late radiation effects in the dog brain: correlation of MRI and histological changes. Radiother Oncol. (2002) 63:107–20. 10.1016/S0167-8140(02)00028-2 12065111

[13] Rahmathulla G Marko NF Weil RJ. Cerebral radiation necrosis: a review of the pathobiology, diagnosis and management considerations. J Clin Neurosci. (2013) 20:485–502. 10.1016/j.jocn.2012.09.011 23416129

[14] Remler MP Marcussen WH Tiller-Borsich J. The late effects of radiation on the blood brain barrier. Int J Radiat Oncol Biol Phys. (1986) 12:1965–9. 10.1016/0360-3016(86)90133-1 3771316

[15] Fuks Z Kolesnick R. Engaging the vascular component of the tumor response. Cancer Cell (2005) 8:89–91. 10.1016/j.ccr.2005.07.014 16098459

[16] Daigle JL Hong JH Chiang CS McBride WH. The role of tumor necrosis factor signaling pathways in the response of murine brain to irradiation. Cancer Res. (2001) 61:8859–65. Available online at: http://cancerres.aacrjournals.org/content/61/24/8859.article-info 11751409

[17] Nordal RA Wong CS. Molecular targets in radiation-induced blood-brain barrier disruption. Int J Radiat Oncol Biol Phys. (2005) 62:279–87. 10.1016/j.ijrobp.2005.01.039 15850934

[18] Nordal RA Nagy A Pintilie M Wong CS. Hypoxia and hypoxia-inducible factor-1 target genes in central nervous system radiation injury: a role for vascular endothelial growth factor. Clin Cancer Res. (2004) 10:3342–53. 10.1158/1078-0432.CCR-03-0426 15161688

[19] Nordal RA Wong CS. Intercellular adhesion molecule-1 and blood-spinal cord barrier disruption in central nervous system radiation injury. J Neuropathol Exp Neurol. (2004) 63:474–83. 10.1093/jnen/63.5.474 15198126

[20] Burger PC Mahley MS Jr Dudka L Vogel FS The morphologic effects of radiation administered therapeutically for intracranial gliomas: a postmortem study of 25 cases. Cancer (1979) 44:1256–72. 10.1002/1097-0142(197910)44:4<1256::AID-CNCR2820440415>3.0.CO;2-T 387205

[21] Panagiotakos G Alshamy G Chan B Abrams R Greenberg E Saxena A. Long-term impact of radiation on the stem cell and oligodendrocyte precursors in the brain. PLoS ONE (2007) 2:e588. 10.1371/journal.pone.0000588 17622341 PMC1913551

[22] Sneed PK Mendez J Vemer-van den Hoek JG Seymour ZA Ma L Molinaro AM. Adverse radiation effect after stereotactic radiosurgery for brain metastases: incidence, time course, and risk factors. J Neurosurg. (2015) 123:373–86. 10.3171/2014.10.JNS141610 25978710

[23] Shaw E Scott C Souhami L Dinapoli R Bahary JP Kline R. Radiosurgery for the treatment of previously irradiated recurrent primary brain tumors and brain metastases: initial report of radiation therapy oncology group protocol (90-05). Int J Radiat Oncol Biol Phys. (1996) 34:647–54. 10.1016/0360-3016(95)02106-X 8621289

[24] Flickinger JC Kondziolka D Lunsford LD Kassam A Phuong LK Liscak R. Development of a model to predict permanent symptomatic postradiosurgery injury for arteriovenous malformation patients. Arteriovenous Malformation Radiosurgery Study Group. Int J Radiat Oncol Biol Phys. (2000) 46:1143–8. 10.1016/S0360-3016(99)00513-1 10725624

[25] Korytko T Radivoyevitch T Colussi V Wessels BW Pillai K Maciunas RJ. 12 Gy gamma knife radiosurgical volume is a predictor for radiation necrosis in non-AVM intracranial tumors. Int J Radiat Oncol Biol Phys. (2006) 64:419–24. 10.1016/j.ijrobp.2005.07.980 16226848

[26] Minniti G D'Angelillo RM Scaringi C Trodella LE Clarke E Matteucci P. Fractionated stereotactic radiosurgery for patients with brain metastases. J Neurooncol. (2014) 117:295–301. 10.1007/s11060-014-1388-3 24488446

[27] Wegner RE Leeman JE Kabolizadeh P Rwigema JC Mintz AH Burton SA. Fractionated stereotactic radiosurgery for large brain metastases. Am J Clin Oncol. (2015) 38:135–9. 10.1097/COC.0b013e31828aadac 23563213

[28] Ruben JD Dally M Bailey M Smith R McLean CA Fedele P. Cerebral radiation necrosis: incidence, outcomes, and risk factors with emphasis on radiation parameters and chemotherapy. Int J Radiat Oncol Biol Phys. (2006) 65:499–508. 10.1016/j.ijrobp.2005.12.002 16517093

[29] Ohtakara K Hayashi S Nakayama N Ohe N Yano H Iwama T. Significance of target location relative to the depth from the brain surface and high-dose irradiated volume in the development of brain radionecrosis after micromultileaf collimator-based stereotactic radiosurgery for brain metastases. J Neurooncol. (2012) 108:201–9. 10.1007/s11060-012-0834-3 22392126

[30] Miller JA Bennett EE Xiao R Kotecha R Chao ST Vogelbaum MA. Association between radiation necrosis and tumor biology after stereotactic radiosurgery for brain metastasis. Int J Radiat Oncol Biol Phys. (2016) 96:1060–9. 10.1016/j.ijrobp.2016.08.039 27742540

[31] Kirkpatrick JP Wang Z Sampson JH McSherry F Herndon JE II Allen KJ. Defining the optimal planning target volume in image-guided stereotactic radiosurgery of brain metastases: results of a randomized trial. Int J Radiat Oncol Biol Phys. (2015) 91:100–8. 10.1016/j.ijrobp.2014.09.004 25442342

[32] Raaphorst GP Malone S Alsbeih G Souhani L Szumacher E Girard A. Skin fibroblasts *in vitro* radiosensitivity can predict for late complications following AVM radiosurgery. Radiother Oncol. (2002) 64:153–6. 10.1016/S0167-8140(02)00076-2 12242124

[33] Barajas RF Chang JS Sneed PK Segal MR McDermott MW Cha S. Distinguishing recurrent intra-axial metastatic tumor from radiation necrosis following gamma knife radiosurgery using dynamic susceptibility-weighted contrast-enhanced perfusion MR imaging. AJNR Am J Neuroradiol. (2009) 30:367–72. 10.3174/ajnr.A1362 19022867 PMC7051401

[34] Forsyth PA Kelly PJ Cascino TL Scheithauer BW Shaw EG Dinapoli RP. Radiation necrosis or glioma recurrence: is computer-assisted stereotactic biopsy useful? J Neurosurg. (1995) 82:436–44. 10.3171/jns.1995.82.3.0436 7861222

[35] Detsky JS Keith J Conklin J Symons S Myrehaug S Sahgal A. Differentiating radiation necrosis from tumor progression in brain metastases treated with stereotactic radiotherapy: utility of intravoxel incoherent motion perfusion MRI and correlation with histopathology. J Neurooncol. (2017) 134:433–41. 10.1007/s11060-017-2545-2 28674974

[36] Dequesada IM Quisling RG Yachnis A Friedman WA. Can standard magnetic resonance imaging reliably distinguish recurrent tumor from radiation necrosis after radiosurgery for brain metastases? A radiographic-pathological study. Neurosurgery (2008) 63:898–903; discussion 904. 10.1227/01.NEU.0000333263.31870.31 19005380

[37] Chao ST Ahluwalia MS Barnett GH Stevens GH Murphy ES Stockham AL. Challenges with the diagnosis and treatment of cerebral radiation necrosis. Int J Radiat Oncol Biol Phys. (2013) 87:449–57. 10.1016/j.ijrobp.2013.05.015 23790775

[38] Hu LS Baxter LC Smith KA Feuerstein BG Karis JP Eschbacher JM. Relative cerebral blood volume values to differentiate high-grade glioma recurrence from posttreatment radiation effect: direct correlation between image-guided tissue histopathology and localized dynamic susceptibility-weighted contrast-enhanced perfusion MR imaging measurements. AJNR Am J Neuroradiol. (2009) 30:552–8. 10.3174/ajnr.A1377 19056837 PMC7051449

[39] Cha S Neuroimaging in neuro-oncology. Neurotherapeutics (2009) 6:465–77. 10.1016/j.nurt.2009.05.002 19560737 PMC5084183

[40] Alexiou GA Tsiouris S Kyritsis AP Voulgaris S Argyropoulou MI Fotopoulos AD. Glioma recurrence versus radiation necrosis: accuracy of current imaging modalities. J Neurooncol. (2009) 95:1–11. 10.1007/s11060-009-9897-1 19381441

[41] Muto M Frauenfelder G Senese R Zeccolini F Schena E Giurazza F Dynamic susceptibility contrast (DSC) perfusion MRI in differential diagnosis between radionecrosis and neoangiogenesis in cerebral metastases using rCBV, rCBF and K2. Radiol Med. (2018) 123:545–52. 10.1007/s11547-018-0866-7 29508242

[42] Kim DY Kim HS Goh MJ Choi CG Kim SJ. Utility of intravoxel incoherent motion MR imaging for distinguishing recurrent metastatic tumor from treatment effect following gamma knife radiosurgery: initial experience. AJNR Am J Neuroradiol. (2014) 35:2082–90. 10.3174/ajnr.A3995 24970548 PMC7965176

[43] Matsusue E Fink JR Rockhill JK Ogawa T Maravilla KR. Distinction between glioma progression and post-radiation change by combined physiologic MR imaging. Neuroradiology (2010) 52:297–306. 10.1007/s00234-009-0613-9 19834699

[44] Zeng QS Li CF Zhang K Liu H Kang XS Zhen JH. Multivoxel 3D proton MR spectroscopy in the distinction of recurrent glioma from radiation injury. J Neurooncol. (2007) 84:63–9. 10.1007/s11060-007-9341-3 17619225

[45] Shah R Vattoth S Jacob R Manzil FF O'Malley JP Borghei P. Radiation necrosis in the brain: imaging features and differentiation from tumor recurrence. Radiographics (2012) 32:1343–59. 10.1148/rg.325125002 22977022

[46] Mehrabian H Desmond KL Soliman H Sahgal A Stanisz GJ. Differentiation between radiation necrosis and tumor progression using chemical exchange saturation transfer. Clin Cancer Res. (2017) 23:3667–75. 10.1158/1078-0432.CCR-16-2265 28096269

[47] Patronas NJ Di Chiro G Brooks RA DeLaPaz RL Kornblith PL Smith BH. Work in progress: [18F] fluorodeoxyglucose and positron emission tomography in the evaluation of radiation necrosis of the brain. Radiology (1982) 144:885–9. 10.1148/radiology.144.4.6981123 6981123

[48] Ricci PE Karis JP Heiserman JE Fram EK Bice AN Drayer BP. Differentiating recurrent tumor from radiation necrosis: time for re-evaluation of positron emission tomography? AJNR Am J Neuroradiol. (1998) 19:407–13. 9541290 PMC8338276

[49] Thompson TP Lunsford LD Kondziolka D. Distinguishing recurrent tumor and radiation necrosis with positron emission tomography versus stereotactic biopsy. Stereotact Funct Neurosurg. (1999) 73:9–14. 10.1159/000029743 10853090

[50] Isselbacher KJ. Sugar and amino acid transport by cells in culture–differences between normal and malignant cells. N Engl J Med. (1972) 286:929–33. 10.1056/NEJM197204272861707 4335317

[51] Terakawa Y Tsuyuguchi N Iwai Y Yamanaka K Higashiyama S Takami T. Diagnostic accuracy of 11C-methionine PET for differentiation of recurrent brain tumors from radiation necrosis after radiotherapy. J Nucl Med. (2008) 49:694–9. 10.2967/jnumed.107.048082 18413375

[52] Park J Byun BH Lim I Kim BI Choi CW Lim SM Comparison of diagnostic performances of F-18 FDG, F-18 FLT, and F-18 FET brain PET/CT for differentiating recurrent glioma from post-treatment change. J Nucl Med. (2014) 55:526 Available online at: http://jnm.snmjournals.org/citmgr?gca=jnumed%3B55%2Fsupplement_1%2F526

[53] Miyake K Shinomiya A Okada M Hatakeyama T Kawai N Tamiya T. Usefulness of FDG, MET and FLT-PET studies for the management of human gliomas. J Biomed Biotechnol. (2012) 2012:205818. 10.1155/2012/205818 22577290 PMC3336213

[54] Galldiks N Stoffels G Filss CP Piroth MD Sabel M Ruge MI. Role of O-(2-(18)F-fluoroethyl)-L-tyrosine PET for differentiation of local recurrent brain metastasis from radiation necrosis. J Nucl Med. (2012) 53:1367–74. 10.2967/jnumed.112.103325 22872742

[55] Rachinger W Goetz C Popperl G Gildehaus FJ Kreth FW Holtmannspotter M. Positron emission tomography with O-(2-[18F]fluoroethyl)-l-tyrosine versus magnetic resonance imaging in the diagnosis of recurrent gliomas. Neurosurgery (2005) 57:505–11; discussion 505–11. 10.1227/01.NEU.0000171642.49553.B0 16145529

[56] Truong MT St Clair EG Donahue BR Rush SC Miller DC Formenti SC. Results of surgical resection for progression of brain metastases previously treated by gamma knife radiosurgery. Neurosurgery (2006) 59:86–97; discussion 86–97. 10.1227/01.NEU.0000219858.80351.38 16823304

[57] Telera S Fabi A Pace A Vidiri A Anelli V Carapella CM. Radionecrosis induced by stereotactic radiosurgery of brain metastases: results of surgery and outcome of disease. J Neurooncol. (2013) 113:313–25. 10.1007/s11060-013-1120-8 23525948

[58] Szeifert GT Atteberry DS Kondziolka D Levivier M Lunsford LD. Cerebral metastases pathology after radiosurgery: a multicenter study. Cancer (2006) 106:2672–81. 10.1002/cncr.21946 16700040

[59] Jagannathan J Bourne TD Schlesinger D Yen CP Shaffrey ME Laws ER Jr. Clinical and pathological characteristics of brain metastasis resected after failed radiosurgery. Neurosurgery (2010) 66:208–17. 10.1227/01.NEU.0000359318.90478.69 20023552

[60] Vecil GG Suki D Maldaun MV Lang FF Sawaya R. Resection of brain metastases previously treated with stereotactic radiosurgery. J Neurosurg. (2005) 102:209–15. 10.3171/jns.2005.102.2.0209 15739546

[61] Perry A 21–Therapy-Associated Neuropathology, Practical Surgical Neuropathology: A Diagnostic Approach. 2nd ed Philadelphia, PA: Elsevier (2018). p. 493–503. Available online at: https://www.elsevier.com/books/practical-surgical-neuropathology-a-diagnostic-approach/9780323449410

[62] Wang YX King AD Zhou H Leung SF Abrigo J Chan YL. Evolution of radiation-induced brain injury: MR imaging-based study. Radiology (2010) 254:210–8. 10.1148/radiol.09090428 20019142

[63] Patel TR McHugh BJ Bi WL Minja FJ Knisely JP Chiang VL. A comprehensive review of MR imaging changes following radiosurgery to 500 brain metastases. AJNR Am J Neuroradiol. (2011) 32:1885–92. 10.3174/ajnr.A2668 21920854 PMC7966021

[64] Kotsarini C Griffiths PD Wilkinson ID Hoggard N. A systematic review of the literature on the effects of dexamethasone on the brain from *in vivo* human-based studies: implications for physiological brain imaging of patients with intracranial tumors. Neurosurgery (2010) 67:1799–815; discussion 1815. 10.1227/NEU.0b013e3181fa775b 21107211

[65] Kim JH Chung YG Kim CY Kim HK Lee HK. Upregulation of VEGF and FGF2 in normal rat brain after experimental intraoperative radiation therapy. J Korean Med Sci. (2004) 19:879–86. 10.3346/jkms.2004.19.6.879 15608402 PMC2816293

[66] Tye K Engelhard HH Slavin KV Nicholas MK Chmura SJ Kwok Y. An analysis of radiation necrosis of the central nervous system treated with bevacizumab. J Neurooncol. (2014) 117:321–7. 10.1007/s11060-014-1391-8 24504500

[67] Levin VA Bidaut L Hou P Kumar AJ Wefel JS Bekele BN. Randomized double-blind placebo-controlled trial of bevacizumab therapy for radiation necrosis of the central nervous system. Int J Radiat Oncol Biol Phys. (2011) 79:1487–95. 10.1016/j.ijrobp.2009.12.061 20399573 PMC2908725

[68] Williamson R Kondziolka D Kanaan H Lunsford LD Flickinger JC. Adverse radiation effects after radiosurgery may benefit from oral vitamin E and pentoxifylline therapy: a pilot study. Stereotact Funct Neurosurg. (2008) 86:359–66. 10.1159/000163557 18854663

[69] Glantz MJ Burger PC Friedman AH Radtke RA Massey EW Schold SC Jr. Treatment of radiation-induced nervous system injury with heparin and warfarin. Neurology (1994) 44:2020–7. 10.1212/WNL.44.11.2020 7969953

[70] Chuba PJ Aronin P Bhambhani K Eichenhorn M Zamarano L Cianci P. Hyperbaric oxygen therapy for radiation-induced brain injury in children. Cancer (1997) 80:2005–12. 10.1002/(SICI)1097-0142(19971115)80:10<2005::AID-CNCR19>3.0.CO;2-0 9366305

[71] Kohshi K Imada H Nomoto S Yamaguchi R Abe H Yamamoto H. Successful treatment of radiation-induced brain necrosis by hyperbaric oxygen therapy. J Neurol Sci. (2003) 209:115–7. 10.1016/S0022-510X(03)00007-8 12686413

[72] Leber KA Eder HG Kovac H Anegg U Pendl G. Treatment of cerebral radionecrosis by hyperbaric oxygen therapy. Stereotact Funct Neurosurg. (1998) 70:229–36. 10.1159/000056426 9782255

[73] Ohguri T Imada H Kohshi K Kakeda S Ohnari N Morioka T. Effect of prophylactic hyperbaric oxygen treatment for radiation-induced brain injury after stereotactic radiosurgery of brain metastases. Int J Radiat Oncol Biol Phys. (2007) 67:248–55. 10.1016/j.ijrobp.2006.08.009 17189073

[74] McPherson CM Warnick RE. Results of contemporary surgical management of radiation necrosis using frameless stereotaxis and intraoperative magnetic resonance imaging. J Neurooncol. (2004) 68:41–7. 10.1023/B:NEON.0000024744.16031.e9 15174520

[75] Rahmathulla G Recinos PF Valerio JE Chao S Barnett GH. Laser interstitial thermal therapy for focal cerebral radiation necrosis: a case report and literature review. Stereotact Funct Neurosurg. (2012) 90:192–200. 10.1159/000338251 22678505

[76] Ahluwalia M Barnett GH Deng D Tatter SB Laxton AW Mohammadi AM Laser ablation after stereotactic radiosurgery: a multicenter prospective study in patients with metastatic brain tumors and radiation necrosis. J Neurosurg. (2018) 4:1-8. 10.3171/2017.11.JNS171273. 29726782

